# Social opportunities and mate preference improve breeding success in Caribbean iguanas

**DOI:** 10.1038/s41598-023-47599-3

**Published:** 2023-11-27

**Authors:** Jeffrey M. Lemm, Meghan S. Martin

**Affiliations:** 1 Conservation Science and Wildlife Health, San Diego Zoo Wildlife Alliance, 15600 San Pasqual Valley Rd., Escondido, CA 92027 USA; 2 PDXWildlife, 5223 SE 41st Ave., Portland, OR 97202 USA

**Keywords:** Animal behaviour, Herpetology

## Abstract

Conservation breeding of West Indian rock iguanas (*Cyclura*) has met with limited success historically. Many facilities witness high levels of aggression and mate incompatibility resulting in failed breeding introductions which often require animals to be separated. This may be due, in part, to lack of knowledge of how mate familiarity and preference affect reproductive outcomes in these species. We investigated whether social exposure during the pre-breeding season influenced copulation success, egg production, and breeding behaviors. Additionally, we examined whether mate preference, as determined by pre-mating dichotomous choice tests, increased these reproductive outcomes. Female rock iguanas that were socialized with males prior to breeding opportunities copulated with familiar males for longer periods of time than females that were not socialized. Socialization opportunities did not alter male reproductive success measurements or breeding behavior. Female rock iguanas introduced for mating to their preferred partners had a higher probability of successful copulations, higher average number of copulations, and less resting behavior during introductions than females mated to non-preferred males. Male mate preference had no effect on reproductive success measurements during mating introductions. These results indicate that socializing animals and providing mate choice opportunities increase breeding success of rock iguanas under managed care.

## Introduction

The success of captive breeding programs often hinges on the ability to recreate natural social structures and behaviors among animals. While these techniques are starting to be adopted more widely for many mammals and birds, the significance of socialization in reptiles’ breeding success has been somewhat overlooked historically which is surprising given the social nature of these reptiles. West Indian rock iguanas (*Cyclura*) are among the most endangered lizards in the world with eight of the ten recognized living species listed as endangered or critically endangered (IUCN 2022), primarily due to loss of habitat and introduced predators^1^. Although ex-situ conservation breeding programs for *Cyclura* have had some success in producing viable offspring in the United States, reliable and successful breeding for the more critically endangered species has been limited^2,3^. Breeding programs and the pairing of rock iguanas are based on the genetics and demographics of proposed rock iguana pairs and the studbooks for the species^2^ with little to no attention given to behavioral compatibility and mate preference.

As social animals, iguanas communicate using visual and chemical cues^4^. *Cyclura* exhibit a diversity of social behaviors in which some species are extremely territorial and others live in large aggregations or social groups^5^. Furthermore, they may recognize and behave differently toward individuals of different dominance rank and often form social groups in which individuals differentiate members of their own group and alter their behaviors toward familiar individuals^6^. Thus, it is likely that familiarity and mate preference play a role in mating success. Indeed, in several species of smaller reptiles, individuals mate based on familiarity with individuals of the opposite sex^7–11^. Mate preference and familiarity have been shown to affect reproductive success in many species including the Columbia Basin pygmy rabbit^12^, the giant panda^13^, house mice^14−16^, mallard ducks^17^, and zebra finches^18^. Within these species, intromission, offspring production, offspring survivorship, and offspring fitness have all been increased by breeding individuals to their preferred partners. However, it is unknown how ubiquitous these findings are across taxa and mate familiarity has not been thoroughly researched in larger lizards such as *Cyclura*. Because conservation breeding facilities often maintain limited numbers of potential breeders at any single location, there is a need to further understand the role of mate preference and familiarity in order to optimize reproductive success.

*Cyclura* mating system can be classified as dominance polygyny, with strong intrasexual competition among males and high variability in male mating success^19,20^. Such systems are well known for driving the evolution of female choice and mate preferences and have been discussed since the late nineteenth century^21^. Mate preference in iguanid lizards may rely on a combination of factors. For example, it is believed that large, dominant males in close proximity to multiple females are the most successful in terms of breeding success (i.e. copulation with one or more females, resulting in viable offspring). These males may attract females based on their body and head size, territory quality and size, ability to monopolize resources, and by number, duration and quality of displays^22–27^. However, there seems to be no consensus on the most important factors for iguana breeding success or if mate preference even exists in these species.

Past research suggests that breeding success among large iguanas is influenced by both preference and familiarity. *Cyclura* exhibit a wide range of social behaviors with communicative displays differing between species and even among populations of the same species^28^. In many species, rock iguanas are territorial in the breeding season and males fight for territories that overlap with female territories or for females who might be passing through their territories^19,26,29,30^. Males often guard females within their territories and mate with numerous females; forced matings are common, especially by smaller males^19,27,30^. Therefore, the potential exists for both males and females to demonstrate a preference. Lemm and Alberts^2^ mention finding opposite sex pairs of Cuban iguanas together outside of the breeding season. Likewise, Wiewandt^19^ found that extended courtship accompanied year-round territoriality among successful male Mona island iguanas. This extended courtship may function to establish and maintain a bond with females visiting or residing in their territory. Extended courtship prior to copulation in desert iguanas may be necessary for establishing familiarity between potential mates and for the physiological facilitation of receptivity in females^31^. Thus, it is likely that mate familiarity also plays a significant role in an individual’s breeding success.

Although the San Diego Zoo Wildlife Alliance (SDZWA) has been one of the more successful conservation breeding programs for rock iguanas, as the animals have matured, or due to studbook-recommended pairing changes, egg fertility has decreased and many animals have become behaviorally incompatible and do not copulate. Traditionally, most rock iguana pairs cannot be housed together annually as males become highly aggressive toward females after the nesting season in July or August. Animals are typically paired between April and May, depending on weather. Pairs are given a week or two to become familiar with one another via shared mesh walls in outdoor pens. However, in the winter, when the animals are housed indoors, they do not have visual access to one another and olfactory access is limited. Because we had some success in breeding compatible *C. pinguis* that were paired annually^32^ and we had used howdy doors (i.e. plastic or mesh panels that allowed neighboring animals visual and olfactory access to one another) to introduce *C. collei* juveniles with success (unpub. data), we hypothesized that socialization during the pre-breeding season may be necessary for successful reproduction in mature adults. In February 2017, howdy doors were installed between all indoor rock iguana enclosures at SDZWA. We investigated whether socialization (given through open howdy door access) prior to breeding introduction of pairs increased breeding success, egg production, and mating behaviors in Caribbean iguanas. Additionally, we measured how socialization impacted mate preference of both male and female rock iguanas through dichotomous choice tests prior to and after socialization opportunities.

## Results

### Effects of socialization on reproductive success and breeding behavior

Iguanas were grouped into socialized pairs or non-socialized pairs. Socialized pairs were given visual and limited tactile and olfactory access to each other prior to mating (see “Methods” section). We investigated whether socialization increased reproductive fitness using GLMMs. Measures of reproductive fitness were higher for rock iguana mate pairs that were given howdy door introductions prior to being paired for breeding (Table 1). All best model GLMMs for the effect of socialization on female–male rock iguana pair reproductive success measurements and behaviors are reported in Table 2. Snout-vent length (SVL) is correlated with clutch size in *Cyclura*^2^, which may impact our results if the female SVL between the socialized and non-socialized group is different. However, a GLMM showed no difference between these two groups in SVL (β = 0.22, Z value = 0.05, p = 0.98).

**Table 1:** Measures of reproductive success for mate pairings of rock iguanas for socialized versus unsocialized pairings.

Reproductive variable	Socialized	Unsocialized
Total mate pairings	10	9
Successful copulation (Y/N)	**80% (N = 8)**	**37.5% (N = 3)**
Avg. number of copulations	2.7 ± 3.3	1.1 ± 1.8
Avg. copulation time per mounting (min)	**8.6 ± 11.6**	**1.0 ± 1.9**
Egg production (Y/N)	30% (N = 3)	11.1% (N = 1)
Avg. number of eggs	2.9 ± 5.1	0.5 ± 1.7
Female preference postsocial	50%	33.3%
Male preference postsocial	60%	62.5%*

Bold indicates significant differences between groups in GLMMs (Table 2).

*Indicates a sample size of 8 because one male did not get a post-social preference test score due to camera failure.

**Table 2:** Best generalized linear mixed models for the effect of socialization of male and female rock iguana pairs on the reproductive response variables and female and male breeding behavior.

Explanatory variable	β	Z value	p value
**Reproductive variable**			
Successful copulation (Y/N)	**2.24**	**2.76**	**0.05**
Number of copulations	1.59	1.48	0.16
Avg. total copulation time (min)	**7.76**	**2.13**	**0.05**
Egg production (Y/N)	1.36	0.98	0.33
Avg. number of eggs	1.79	1.29	0.23
Female preference postsocial (Y/N)	− 0.69	− 0.74	0.47
Male preference postsocial (Y/N)	− 0.11	− 0.11	0.91
**Female behavioral variable (reference group is Howdy)**			
Social display	− 1.21	− 0.88	0.39
Proximity	0.01	0.13	0.89
Olfactory communication	− **4.45**	− **2.84**	**0.005**
Feed	0.07	1.32	0.19
Basking	− 0.27	− 1.87	0.06
Resting	− **1.87**	− **4.86**	** < 0.001**
**Male behavioral variable**			
Social display	− 0.04	− 0.04	0.97
Proximity	− 0.08	− 1.84	*0.08*
Olfactory communication	− 2.55	− 1.19	0.24
Feed	− 0.68	1.39	0.18
Basking	0.20	0.58	0.57
Resting	− 1.14	− 0.68	0.50

Animal ID was included as a random factor. Bold indicates significant differences between groups in GLMMs p ≤ 0.05.

Significant values are in italics.

Pairs that were allowed visual and olfactory access through howdy doors prior to mating (i.e. socialized) had significantly more introduction success and longer average copulation times than pairs that were not allowed socialization access (Tables 1, 2). While average total number of copulations, egg production, and average number of eggs produced were almost twice as many for socialized pairs (Table 1), these differences were not significant, likely because of low sample sizes (Table 2). When given dichotomous choice tests after socialization, more females showed a preference toward males they had been socialized with versus males that they had not been socialized with, but this difference was not significant (Table 1). Howdy introductions had no effect on male preference (Table 1).

**Figure 1:**
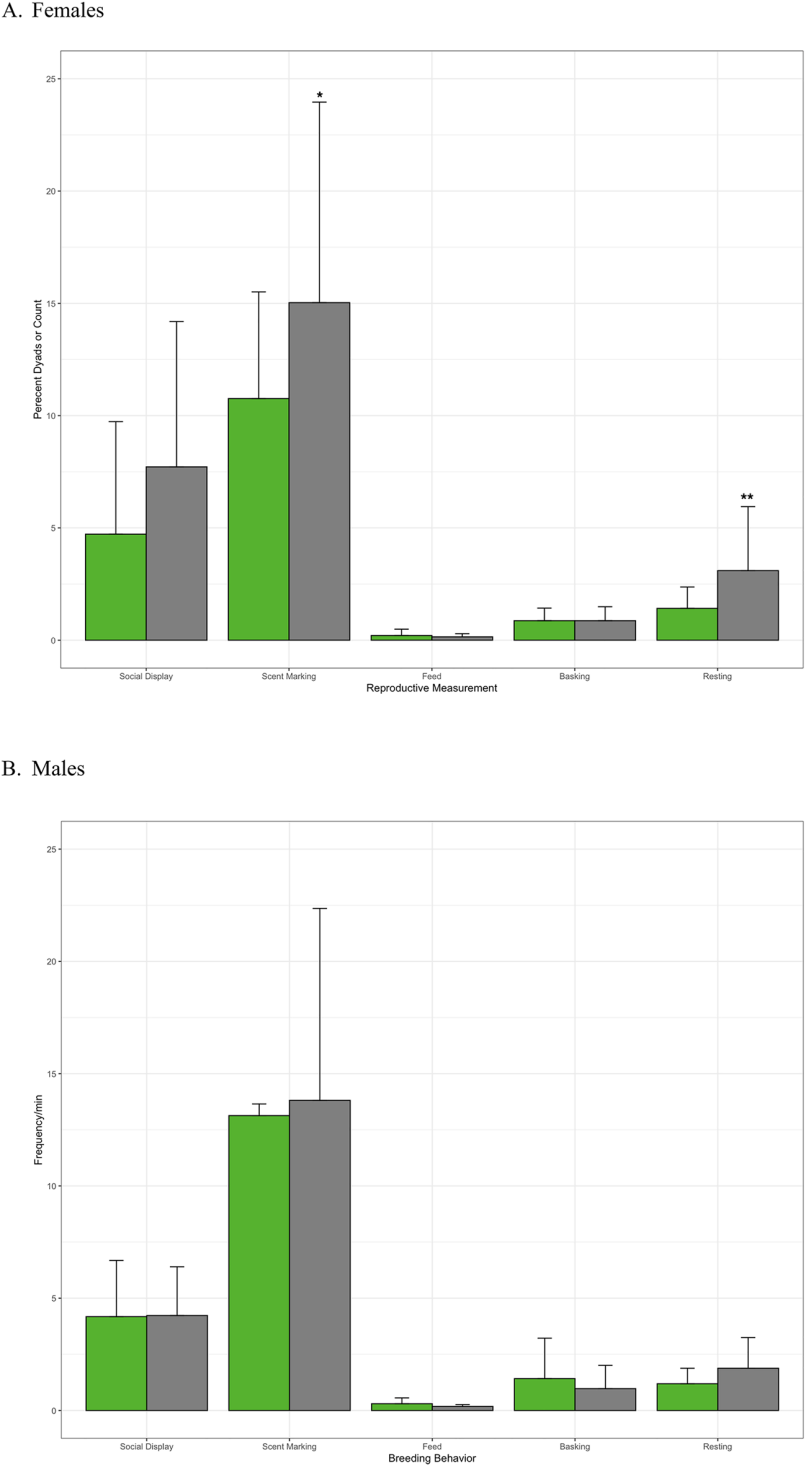
Frequency of breeding behavior for socialized (green) and unsocialized (gray) female (**A**) and male (**B**) rock iguanas during mating introductions. Error bars represent standard deviation.

Female rock iguanas that were socialized with their potential breeding partners prior to mate introductions had significantly less olfactory communication and resting behaviors when the animals were paired for breeding (Table 2, Fig. 1). Whereas male rock iguanas that were socialized with their potential breeding partners prior to mate introductions did not have significantly different behaviors during breeding than animals not socialized prior to mating (Table 2, Fig. 1).

### Effects of mate preference on reproductive success and breeding behavior

Iguanas were tested for mate preference prior to socialization in the breeding season and post-socialization. We investigated whether pre-socialization mate preference increased reproductive fitness measurements using GLMMs. Measures of reproductive fitness were higher for female rock iguanas that were paired with their preferred mates as determined by the pre-social dichotomous choice test (Table 3). A GLMM showed no difference between SVL length between preferred and non-preferred groups when investigating female preference (β =  − 0.23, Z value =  − 0.05, p = 0.96) and male preference (β = 5.13, Z value = 0.89, p = 0.40).

**Table 3:** Measures of reproductive success of female and male rock iguanas introduced during breeding season with preferred versus non-preferred mates.

Reproductive variable	Preferred	Nonpreferred
**Female preference**		
Total mate pairings	11	8
Successful copulation (Y/N)	**72.7% (N = 8)**	**37.5% (N = 3)**
Number of copulations	**3.09 ± 3.11**	**0.38 ± 0.52**
Avg. total copulation time (min)	51.95 ± 107.58	2.13 ± 3.98
Egg production (Y/N)	27.3% (N = 3)	14.3% (N = 1)
Avg. number of eggs	1.82 ± 3.37	1.75 ± 4.95
Male mass (kg)	5.33 ± 1.27	5.42 ± 1.12
Male SVL (mm)	482.73 ± 37.71	483.75 ± 28.76
Male tail length (mm)	590.91 ± 48.26	575.00 ± 60.71
Male age (years)	21.09 ± 7.06	21.88 ± 8.85
**Male preference**		
Total mate pairings	11	7
Successful copulation (Y/N)	45.5% (N = 5)	85.7% (N = 6)
Avg. number of copulations	2.08 ± 3.34	1.71 ± 1.25
Avg. total copulation time (min)	44.58 ± 104.85	7.64 ± 7.88
Egg production (Y/N)	27.3% (N = 3)	14.3% (N = 1)
Avg. number of eggs	1.67 ± 3.26	2 ± 5.29
Female mass (kg)	2.84 ± 1.37	3.1 ± 1.67
Female SVL (mm)	373.33 ± 54.49	382.86 ± 55.89
Female tail length (mm)	480.00 ± 75.20	502.86 ± 69.45
Female age (years)	16.83 ± 7.78	21.0 ± 10.20
**Mutual mate preference**		
Total mate pairings	7	11
Successful copulation (Y/N)	71.4% (N = 5)	54.5% (N = 6)
Avg. number of copulations	**4 ± 3.95**	**0.82 ± 0.87**
Avg. total copulation time (min)	*79.43* ± *139.47*	*2.95* ± *2.50*
Egg production (Y/N)	27.3% (N = 3)	14.3% (N = 1)
Avg. number of eggs	2.86 ± 4.08	1.27 ± 4.22

Bold indicates significant differences between groups in GLMMs p ≤ 0.05. For mutual mate preference, the preferred group is defined as when both the male and female preferred each other in a dichotomous choice test, the nonpreferred group is all other pairings (both male and female nonpreferred, female prefers male but male does not prefer the female and vice versa).

Significant values are in italics.

**Figure 2:**
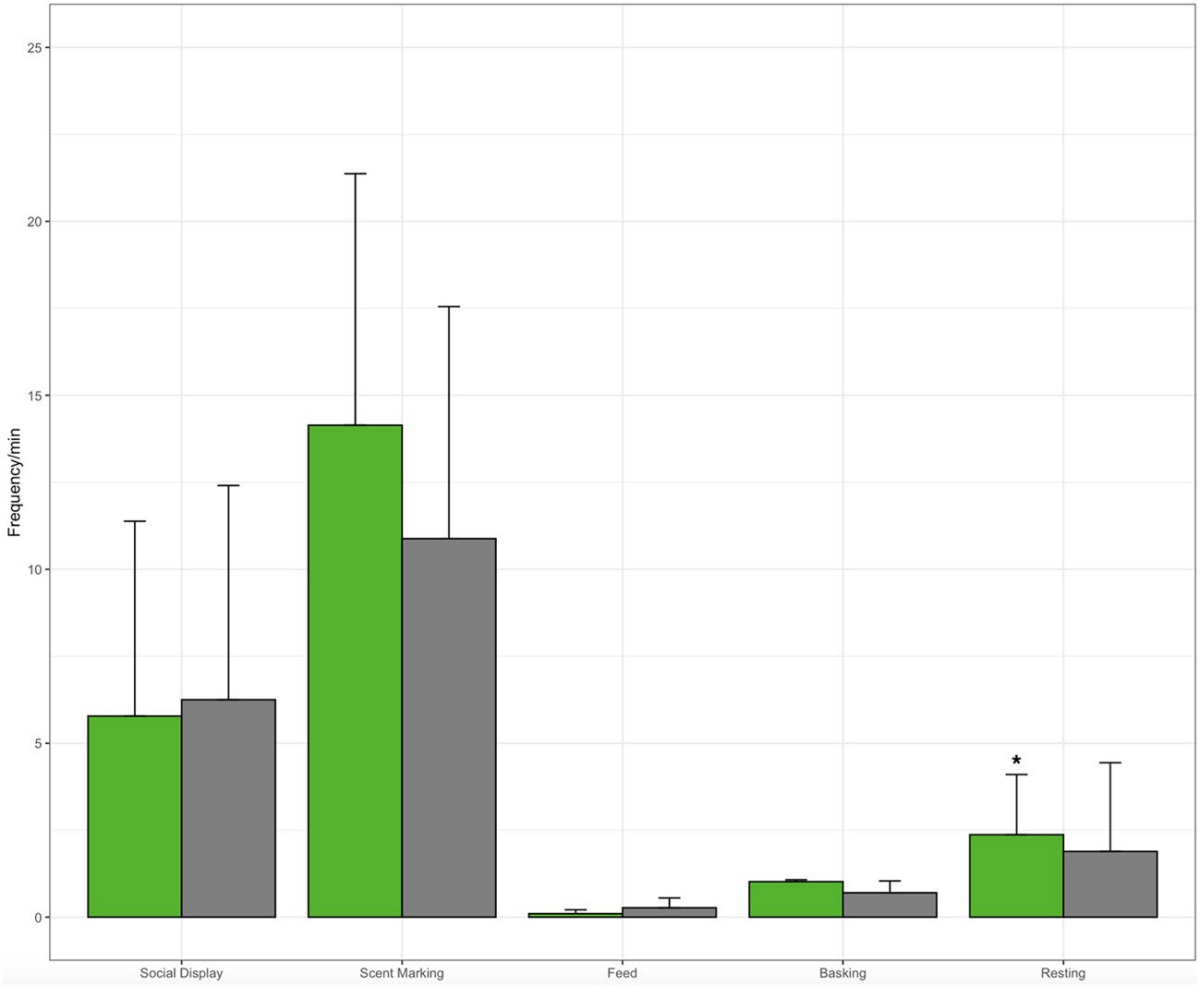
Behaviors during breeding introductions for female rock iguanas mated with either their preferred (green) or nonpreferred (gray) partner. Error bars represent standard deviation.

All best model GLMMs for the effect of preference on female and male rock iguana reproductive success measurements and behaviors are reported in Table 4. Females mated to their preferred partner had significantly more copulation success and average number of copulations, but not average total copulation time, egg production, nor average number of eggs for the breeding season than females mated to their non-preferred mate (Tables 3, 4). Females did not seem to prefer males based on male weight, snout-vent length, tail length, or age (Table 4). Female rock iguanas that preferred their potential breeding partners had significantly fewer resting behaviors during mating introductions but were no different in all other measured behavioral categories than females mated to their non preferred partners (Table 4, Fig. 2).

**Table 4:** Best generalized linear mixed models for the effect of male and female rock iguana mate preference on the reproductive explanatory variables and female and male breeding behavior during breeding introductions.

Explanatory variable	Female			Male		
	β	Z value	p value	β	Z value	p value
**Reproductive variable**						
Successful copulation (Y/N)	**21.84**	**2.34**	**0.02**	− 1.97	− 1.59	0.11
Number of copulations	**2.45**	**2.38**	**0.03**	1.31	0.62	0.54
Avg. total copulation time (min)	6.19	1.51	0.15	2.26	0.49	0.63
Egg production (Y/N)	1.53	0.71	0.48	1.31	0.62	0.54
Avg. number of eggs	0.79	0.57	0.59	1.953	1.31	0.22
**Behavioral variable**						
Social display	− 2.86	− 1.2	0.24	**2.02**	**1.89**	**0.05**
Proximity	0.04	0.59	0.56	− 0.02	− 0.45	0.66
Olfactory communication	− 4.27	− 1.42	0.17	**5.48**	**1.91**	**0.05**
Feed	0.10	0.59	0.57	0.12	1.57	0.14
Basking	− 0.18	− 0.72	0.48	0.64	1.55	0.14
Resting	− **2.68**	− **2.91**	**0.05**	0.47	0.96	0.35

Animal ID was included as a random factor. Bold indicates significant differences between groups in GLMMs p ≤ 0.05.

**Figure 3:**
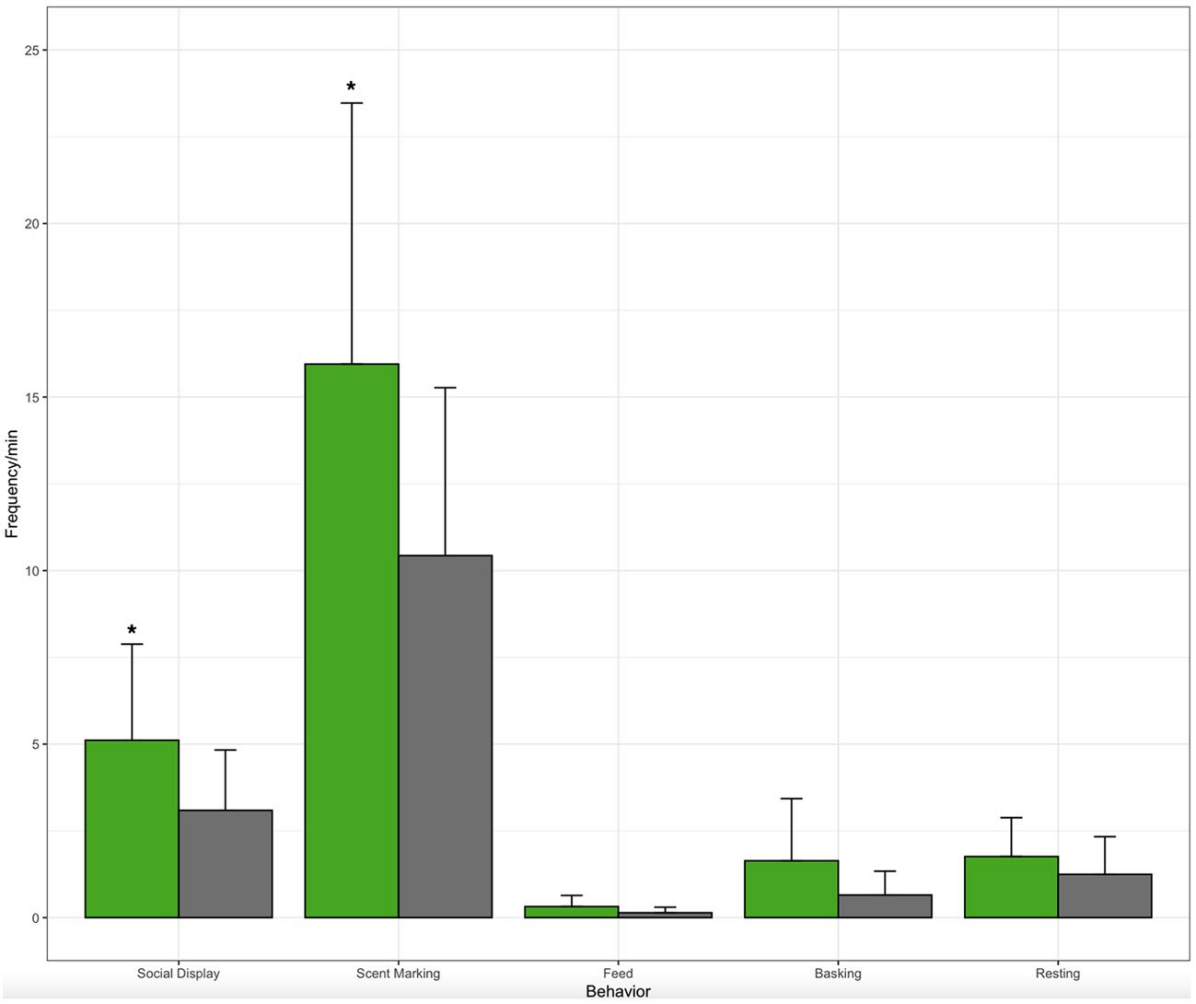
Behaviors during breeding introductions for male rock iguanas mated with either their preferred (green) or nonpreferred (gray) partner.

Reproductive success measurements for male rock iguanas mated to their preferred partner were not significantly different than males mated to non-preferred females (Table 4). Males did not seem to prefer females based on female weight, snout-vent length, tail length, or age. Male rock iguanas that preferred their potential breeding partners had significantly more social displays and Olfactory Communication than males that were introduced to their non-preferred partner (Table 4, Fig. 3).

Pairings that had mutually preferred partners had significantly more copulations than pairings where one partner did not prefer the other partner or where neither pairing preferred the other (Tables 3, 5). Mutually preferred pairings also had a trend toward a longer copulation time.

**Table 5:** Best generalized linear mixed models for the effect of rock iguana mutual mate preference on the reproductive explanatory variables.

Explanatory variable	Mutual mate preference		
	β	Z value	p value
Successful copulation (Y/N)	1.22	0.77	0.44
Number of copulations	**3.09**	**2.84**	**0.01**
Avg. total copulation time (min)	7.85	1.87	0.08
Egg production (Y/N)	3.05	0.62	0.54
Avg. number of eggs	1.95	1.04	0.32

Female ID was included as a random factor. Bold indicates significant differences between groups in GLMMs p ≤ 0.05.

### Mate preference and consistency between pre- and post-socialization choice test

A binomial test investigating the mate preference consistency between the pre- and post-socialization dichotomous choice test for female rock iguanas found that 68.4% of females maintained their preference between the two time periods (N = 19, p = 0.17) which was not significantly different from chance.

A binomial test investigating the mate preference consistency between the pre- and post-socialization dichotomous choice test for male rock iguanas found that 66.6% of males maintained their preference between the two time periods (N = 18, p = 0.24) which was not statistically significant.

### The importance of socialization versus preference

Socialization groupings were randomly assigned, and mate preference was not determined prior to data analysis. Thus, it is difficult to completely disentangle preference from socialization in this study as post-hoc analysis indicated that preferred mates were not balanced across socialization experimental groups. In the socialized group of ten females, eight initially showed a preference for the male they were eventually paired with during the pre-socialization dichotomous choice test (80%). However, during the post-socialization choice test, three of these females (37.5%) reversed their preference. The other two females in the socialization group were socialized with their non-preferred male as determined by the pre-socialization test and did not flip their preference in the post-socialization mate choice tests (100%). Of the nine females in the non-socialization group six did not initially show a preference for that male during the pre-socialization mate choice test (66%). Among these females, three reversed their preference and ended up selecting the male they previously did not prefer after socialization in the post-socialization mate choice trial (37.5%). The remaining three females in the non-socialization group showed a consistent preference for the males in both the pre- and post-socialization tests (100%).

All best model GLMMs determining the effect of socialization and mutual mate preference on female and male rock iguana reproductive success measurements are reported in Table 6. While both factors were significantly correlated with the number of copulations, average total copulation time, and egg production, the preference factor had a larger effect size for all the reproductive variables.

**Table 6:** Best generalized linear mixed models for the effect of socialization and mutual mate preference on rock iguana reproductive explanatory variables.

Explanatory variable	Preference			Socialization		
	β	Z value	p value	β	Z value	p value
Reproductive variable						
Successful copulation (Y/N)	2.64	1.19	0.24	3.21	1.62	0.11
Number of copulations	**3.46**	**3.59**	**0.003**	**2.12**	**2.42**	**0.035**
Avg. total copulation time (min)	**9.81**	**2.66**	**0.018**	**9.43**	**2.61**	**0.019**
Egg production (Y/N)	**35.72**	**2.20**	**0.028**	**21.57**	**2.09**	**0.037**
Avg. number of eggs	2.12	1.29	0.23	2.07	1.46	0.19

Male and Female ID were included as a random factor.

Significant values are in bold.

## Discussion

Zoological facilities have become conservation breeding centers for many species, including reptiles and amphibians^33–35^. For smaller species such as frogs, single zoos can often house large numbers of animals and provide ample mate choice opportunities. However, for larger species such as rock iguanas, space is severely limited, zoos can rarely house more than a few animals at each facility, and pairs are often chosen based on their genetic profiles through their respective studbooks. While this “Noah’s ark” method of mate pairing is efficient and necessary for small populations, it removes the potential for individuals to choose their own mates and most breeding programs lack a system which gives the animals a choice of mates.

To confound mate pairings, rock iguanas are often kept in habitats much smaller than what the animals are accustomed to in the wild, often resulting in compatibility issues among rock iguana pairs that can lead to separation outside of the breeding months. Additionally, examining mate preference in founding pairs may be unfeasible and extremely resource-intensive^36^. This makes breeding season pairings difficult, and in some cases, the smaller females may be harmed by the larger males when they are brought into contact with one another. Thus, mate pairing methods that reduce aggression and increase compatibility are sorely needed and often require large enclosures, which is difficult for many facilities to facilitate.

This study examined how socialization opportunities of potential mate pairs and mate preference affected reproductive success of endangered rock iguanas. As a study on critically endangered Caribbean rock iguanas, it is not without its limitation which means that our results should be interpreted with caution. Sample sizes were severely limited as was the ability to balance our experimental design between socialization of animals and mate preference. These two factors are confounded in our study, but our in-depth analysis implies both factors increase reproductive success of rock iguanas, but socialization appears to have a greater impact than preference. While individual sample sizes were low, we logged extensive behavioral observations per individual, thus, we feel confident that our results, reflected over a longer period of time, helped to identify common patterns or factors that influence behavior across individuals. Our results indicate that rock iguana conservation breeding programs should give mate choice tests and provide socialization opportunities to rock iguanas prior to breeding introductions.

### Effects of howdy door socialization on reproductive success and breeding behavior

In this study we found that female Caribbean rock iguanas that were socialized with males prior to breeding opportunities copulated for longer periods of time with those males and showed fewer resting behaviors than females that were not socialized. Socialization opportunities did not alter male reproductive success measurements or breeding behavior. Additionally, socialized pairs had almost double the average total number copulations, egg production, and average number of eggs produced than unsocialized pairs. Even though these differences were not significant (likely due to low sample sizes) they suggest that socialization may greatly improve eventual reproductive outcomes.

Our findings on increased reproductive fitness with familiarity are consistent with studies on other lizard species. For example, female Common lizards (*Zootoca vivipara*) treated with corticosterone only mated with familiar males^37^ and Shingle-back skink pairs (*Tiliqua rugosa*) that retained the same partners over time mated earlier in the year than other pairs, which is believed to equate to higher reproductive fitness^11^. This type of long-term familiarity may also be present in *Cyclura* as well. In the wild, some male Mona Island iguanas (*Cyclura stejnegeri*) and Cuban iguanas (*C. n. nubila*) have been documented with females year round^2,19^ and in captive rock iguanas some authors believe that pairs will form long-term bonds when housed together over long periods of time^38^. Increased reproductive success with familiar mates is also well documented in other species^12,14,39^ and likely makes pairs more efficient (e.g. in cases of biparental care) and more coordinated in reproductive behaviors. Familiarity may also function to establish and maintain a bond between pairs and to facilitate receptivity in the female prior to breeding attempts^31^. Our behavioral findings are consistent with previous literature and suggest that receptivity increased for female Caribbean rock iguanas after socialization opportunities.

We observed that socialized female rock iguanas showed significantly less olfactory communication and resting behaviors than females that were not socialized with potential breeding partners. Scent marking in iguanas (via femoral pore secretions and feces) serve as chemical signals to potentially communicate territory, reproductive condition, and health and body condition of the signaler^2,4^. Discrimination between familiar and unfamiliar conspecific lizards by chemical signals has been suggested in several lizard species, including iguanas^40^. Socialized females may reduce their scent marking behaviors in the vicinity of males they were socialized with due to the lack of visual barriers between the proposed mates, enabling visual signaling and reducing the need for chemical signals in close contact. Female desert iguanas that are associated with males have less active glands, suggesting that it may be advantageous for a female to stop olfactorily advertising her presence to other males^4^. The lack of resting behaviors seen when females were paired with familiar males may indicate their willingness and motivation to breed with those males. Female iguanas of several species become more active in the breeding season and visit the territories of potential mates^25,41^. In the current study, it was common to see females moving in and around males in the breeding season, as well as resting near them or even basking on top of them, showing fewer aggressive behaviors.

Male rock iguanas that were socialized with their potential breeding partners prior to mate introductions did not have significantly different behaviors than animals not socialized prior to mating, indicating that males did not alter behaviors towards females they have been socialized with versus not. There was a trend for increased male proximity to potential mates within socialized pairs, although this may be a byproduct of receptivity of females for mating. The fact that females were active and often approached socialized males when housed with them may have been a cue for males to try to mate. Female lizards that show approach and retreat behaviors towards males in some lizard species often heighten male interest^42^. However, it is possible that males are choosing females based on female traits or behaviors that are difficult to quantify or are largely consistent across females during reproductive events^42^.

### Effects of mate preference on reproductive success and breeding behavior

In this study, female rock iguanas that were introduced to their preferred partners for mating, as determined by pre-mating dichotomous choice tests, had a higher probability of successful copulations, higher average number of copulations, and less resting behavior during introductions than females mated to non-preferred males. Females appeared to be more receptive to a preferred male’s mating behavior allowing males increased copulation success and longer copulation times. Males often did not need to overcome females physically and had an easier time grasping the nuchal crests of the females for breeding. In contrast, females mated to males they did not prefer spent much of their time hiding from aggressive males and when the males would attempt to copulate forcefully, females would struggle to get free, thereby reducing the overall copulation times. Forced copulations are common in iguanas, particularly in smaller males that do not hold attractive territories or display as much as larger males^19,27,30^. Our data suggest that preferred males likely do not have to force copulations resulting in higher copulation success.

Mate preference in lizards often exhibits plasticity and may change depending on body condition^43^, personality traits, predation risks^44^, and stress levels^37^. Our results also indicated some plasticity in regard to mate preference in rock iguanas. About one-third of our animals “flipped” preference after socialization but the direction of flipping preference was not consistent (i.e. some non-preferred animals became preferred and vice versa). Additionally, our experimental design did not disentangle mate preference due to seasonal effects (breeding vs. non-breeding) versus our socialization experimental treatment (pre-socialization vs. post-socialization). Future studies should investigate preference more directly without the confound of socialization in these species. In the past, evidence for female mate preference or choice in lizards was thought to be rare^45,46^. More recent literature suggests that female lizards prefer males based on morphology, color pattern, displays, and that chemical senses of female lizards may play an important role as well^47^. Past studies of female preference in lizards have failed to adequately document female preferences or suggest that female choice may be based on multiple male characteristics^48^. Chemical signals probably play a large role in female mate preference and female choice based on pheromones and femoral pore size has been documented in several lizard species^45,49^ including *Cyclura*^26^. Female choice can be complex, highly plastic, and may depend on social environment, ecological conditions, or individual experience^50^. Female preference for mates is not uncommon in other taxa, and in breeding programs for a large variety of species, including insects, fish, birds, and mammals, females that mated with preferred partners increased a variety of reproductive success measurements when compared to assigned mate pairings^51^.

In this study, preferred males did not differ in size (SVL, tail length or mass) or in age from non-preferred males. This is surprising as the ecology of large iguanids would indicate female preference likely exists in the wild, as males fight for high quality territory^26^ and females likely choose males based on size, territory quality or display rates, and well-developed femoral glands^25,26^. In marine iguanas, females have been shown to copulate more with the male that showed the highest display rate of all males visited by a female^25^ and the total number of copulations a male received was strongly associated with head-bob rate but not body mass^52^. In Cuban iguanas, the largest, highest-ranking males also had the greatest display rates^26^. This is consistent with our findings that males showed an increase in social display behaviors and chemical signaling behaviors (i.e. Femoral Pore Drag, Face Rub, Vent Drag, Tongue Flicking, and Defecate; Supplemental Table 1) toward preferred females. However, male preference had no effect on any eventual reproductive success measurements. Preferred females also did not differ from non-preferred females in physical size measurements (SVL, tail length or mass) or their age. These data indicate that males have a greater preference for certain females and will increase their social displays to attract that female but male preference does not appear to impact reproductive success unless females also prefer those males. Because most iguana males in the wild will mate with nearly any female that visits his territory^26,41,52^ it seems likely that the simple act of a female in close proximity to a male would result in increased social and chemical communication behaviors. Our study shows that these behaviors increase even more towards preferred females. It is unclear whether male preference is based on other female physical characteristics, chemical signals, or bimodal female behavior. The possibility that mate choice is based on multiple cues has received increased attention in recent years^53^ and is likely the case in male iguanas as well.

## Conclusions

Our findings indicate that conservation breeding managers should perform a dichotomous choice test, when possible, to determine preference before the breeding season and socialize rock iguanas that show mutual mate preference for several months prior to mate introductions. Preference does not seem to differ between the two time periods we tested (prior to socialization and after) so testing early in the season to determine which male should be socialized would provide for longer periods of familiarization. Though this finding is confounded by our socialization treatment. Because courtship and mating typically occur in May–July, we recommend managers perform mate preference tests between potential partners in March or April. After preference is determined, the preferred male should be socialized with his potential female via howdy doors from the time preference is determined until the animals are paired for breeding.

While both preference and socialization were significantly correlated with increased reproductive output, the preference factor had a slightly larger effect on the number of copulations, average total copulation time, and egg production. The effect of preference on reproductive success was evaluated using the pre-socialization dichotomous choice test (pre-breeding season) because we deemed this time period to be most accessible to the majority of conservation breeding centers for assessing mate preference and relocating animals. We would advocate for conservation breeding programs of *Cyclura* to incorporate both socialization and preference. Realistically, many programs may not have the number of animals, facility size, and/or resources to provide mate socialization and choice. Hence, it is crucial for SSPs and breeding programs to reconsider the existing approach and transition towards a centralized hub for conservation breeding programs. In previous discussions, we have proposed a system in which all animals are transferred to a central breeding facility—the breeding “hub”. There, they would be carefully matched with genetically compatible partners through choice tests before being selectively distributed as successful breeding pairs, as required, for educational and ambassadorial purposes. This approach would optimize the efficiency and effectiveness of conservation efforts.

If facilities are only able to apply one of the two factors investigated in this paper (socialization or mate preference), our results suggest that preference may have a slightly more positive benefit over socialization. However, it is likely that if facilities lack the ability to provide choice, increased reproductive outcomes are possible through socialization alone, particularly when animals are provided with large enclosures. In our experience of over 25 years of breeding *Cyclura,* we suggest facilities build enclosures with multiple sight barriers that allow for physical separation of pairs. If pairs can coexist peacefully, they should be allowed to remain together throughout the year when possible, in order to increase their familiarization. It is our hope that incorporating these recommendations will greatly improve the reproductive success in *Cyclura* conservation breeding programs further increasing the chances of recovering these species from the brink of extinction.

## Methods

### Study site and subjects

This project took place at the Kenneth and Anne Griffin Reptile Conservation Center, a 2500-square foot off-display facility at the San Diego Zoo Safari Park in Escondido, California. Study animals included seven Jamaican iguanas (*Cyclura collei;* two ♂, five♀), six Grand Cayman iguanas (*C. lewisi*; four♂, two♀), one Cuban iguana (*C.n.nubila*; one♂, 0♀) and five Anegada Island iguanas (*C. pinguis*; three♂, two♀). Animals were filmed and recorded daily using two Swann NVR8-7300 8-channel camera/DVR packages.

Because we had limited sample sizes for each species and because some rock iguana species have been shown to interbreed^27,54^ or exhibit relatively similar social displays (J. Lemm, pers. obs.), we housed animals in an alternating sex pattern (male, female, male, female, etc.) to perform dichotomous choice tests. The single male Cuban iguana was not paired with females of other species, but he was used for behavioral components of this study. For the final data set, there were only two occurrences where cross-species dichotomous choice tests were performed. In both instances, the female preferred the male in both the pre- and post-dichotomous choice test and the males were not socialized during the Howdy Socialization period (see below). We performed data analysis on eight focal females repeated across two years. Due to the death of some animals, our final data set had 10 socialized mate dyads (open howdy windows) and nine non-socialized mate dyads (closed howdy windows; see below).

The Kenneth and Anne Griffin Reptile Conservation Center was built specifically for research and ex-situ conservation breeding of West Indian rock iguanas (*Cyclura*) and consisted of 20 indoor/outdoor enclosures, a kitchen, and a nursery. The building was kept at ambient temperatures between 25.6 and 30 °C and humidity varied from 60 to 95%, depending on time of year, cleaning and humidity control routines, and the humidity of outdoor conditions. Each enclosure had a soil depth of one meter in both the indoor and outdoor areas to facilitate burrowing and nesting and enclosures were planted with native Caribbean plants, *Hibiscus*, and various species of palms. Animals received natural sun in the outdoor pens or through UV-transparent windows and skylights. All animals had clean water available at all times and two basking areas. One basking area provided additional UV light (Zoo Med 160 W Powersun UV (Zoo Med Lab, Inc, San Luis Obispo, CA, USA) in 2018 and Zilla Tropical 25 UVA/UVB 20 W (Zilla Products, Central Aquatics, Franklin, WI, USA) in 2019) and the other basking area provided no UV light (Zilla 250 W ceramic heat emitters in 2018 and 550 W Fostoria infrared quartz heaters (TPI corp, Johnson City, TN, USA) in 2019). Basking areas were allowed to reach surface temperatures up to 54.5 °C. Basking areas that emitted light were turned off at night and were maintained on a schedule that matched the natural light cycle throughout the year. Animals were fed daily and each animal received up to 1.0 kg of food, 98% of which consisted of greens (collard, mustard, chard, dandelion, kale, bok choy and escarole). The other 2% of the diet consisted of grated fruits and vegetables (various root vegetables, such as carrots and sweet potatoes) squash, apples and papayas. Twice a week, animals were each fed 15.0 g of diced green beans with their meals. *Hibiscus* flowers were fed as treats up to three times per week in spring and summer (two flowers per animal).

**Figure 4:**
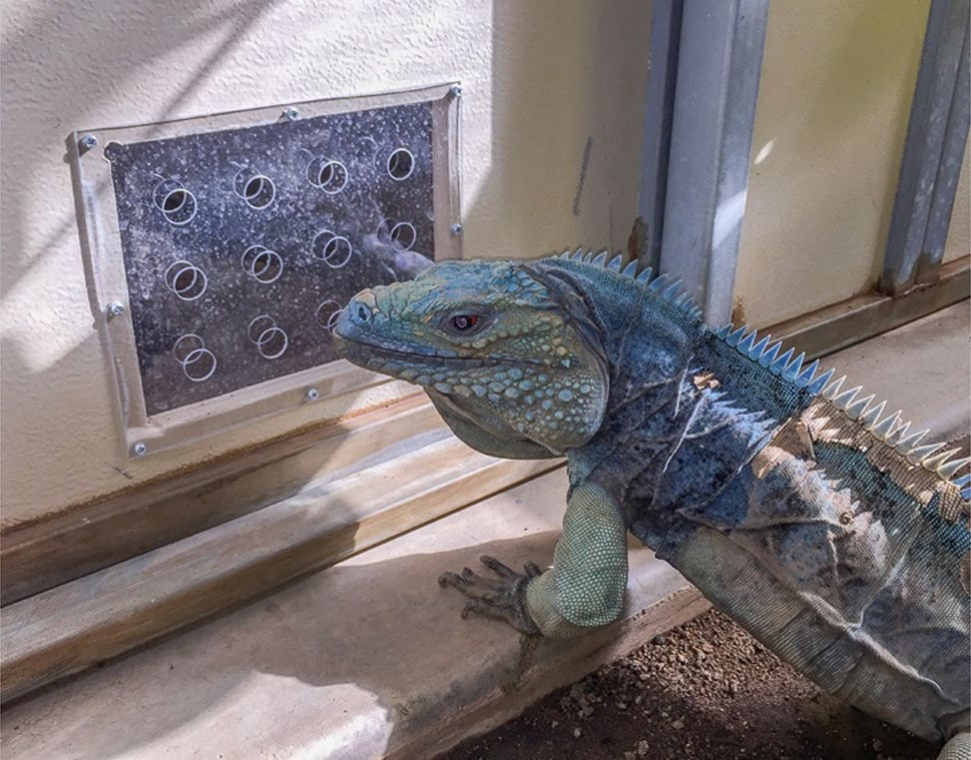
Photo detail of the acrylic howdy doors used to socialize rock iguanas. A Grand Cayman iguana (*C. lewisi*) is pictured.

There were 10 enclosures on either side of the facility (20 total) divided by a long keeper hallway that provided feeding and cleaning access. One enclosure remained empty throughout the study (GRCC-17). Adult enclosures featured an outdoor area measuring 1.83 m × 2.44 m, and indoor enclosures measured 1.83 m × 3.05 m. Both indoor and outdoor enclosures measured 2.4 m high. The indoor and outdoor enclosures were connected via a small door (0.36 × 0.50 m) that remained open during warm weather from May–October, depending on conditions outside. Doors on both the right and left sides of each enclosure (except for the end enclosures, which only had one side door connecting to an adjacent enclosure) allowed access to neighboring pens during the breeding season. These introduction doors measured 0.6 × 1.0 m and featured an acrylic howdy door in the center, measuring 0.31 × 0.20 m. Each howdy door contained up to 12 holes, each measuring 0.025 m in diameter (Fig. 4). The howdy doors enabled neighboring animals to gain visual and chemical access to one another when the doors were uncovered. When a howdy door was covered, a large magnet was attached to the metal frame of the howdy door, covering the acrylic window. These howdy doors were the only areas where rock iguanas had visual access to each other when they were indoors because of the solid wall construction. Outdoor enclosures were divided only by heavy gauge wire mesh and animals were not allowed outdoors during the pre-breeding experimental protocol period and all howdy windows were blocked during this time periods as well (September to April). The howdy door detail can be seen in Fig. 1. Each enclosure was partitioned into two zones to facilitate behavioral scoring, creating left and right sections within the pen.

### Behavioral observations and study protocol

Our overarching goal was to determine if socialization prior to the reproductive season improves reproductive outcomes for captive iguanas. As a secondary goal, we wanted to test whether allowing animals to choose their preferred partners further enhances outcomes. Thus, our study involved two kinds of manipulation: (1) an extended socialization period using ‘howdy doors’, and (2) dichotomous choice tests repeated two times (pre- and post-socialization) each for a period of 24 h.

**Figure 5:**
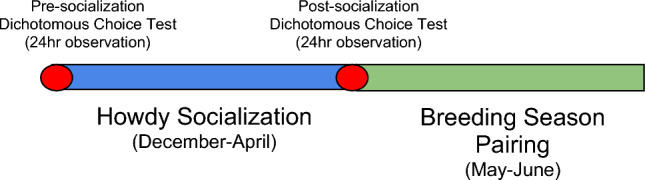
Schematic of research timeline for Caribbean rock iguanas. Average frequency of ethogram behaviors was obtained for 24 h in both a pre- and post-socialization dichotomous choice test (red dots) to determine mate preference. Animals were divided into socialized (had visual and olfactory contact) and non-socialized groups (no visual and olfactory contact) from December to April prior to the breeding season (blue bar). Males and females were then paired for breeding introductions from May to June (green bar).

Animal behaviors during these tests were scored according to a newly defined behavioral ethogram (Supplemental Table 1) to score observational periods defined below (Fig. 5). We grouped behaviors based on a combination of weightings from a Principal Component Analysis (PCA; data not shown) and the experience of experts familiar with rock iguana breeding and behavior (gray rows in Supplemental Table 1). We summed frequencies across each observation of the following behavioral categories (gray rows in Supplemental Table 1): Conspecific investigation, breeding behavior, nesting behavior, olfactory communication behavior, contact aggression, social display behavior, feed, rest, basking behavior, keeper interactions, not visible, and other. Rock iguana behavior was scored manually for all occurrences of the listed behaviors (frequency) through visual inspection of the taped recordings. We pooled species for analysis as all *Cyclura* species share similar behavior and social behavior in one species typically elicits a behavioral response from another species (pers. obs.).

Animals were moved into experimental position (either “socialized” or “not socialized”) for the research season in September to November prior to the pre-socialization trials commencing. This study’s definition of socialization and preference was limited to the current breeding year. However, since both male and female iguanas had been used in previous breeding plans and were repeated across years, it was possible for all animals to be familiar with the recommended breeding partners.

We performed behavioral observations across four distinct trials with the following objectives as follows:

1. *Pre-socialization dichotomous choice trial* One female housed between two males and scored for a single day (0700 to 0700 the following morning) with all howdy doors open on both sides of the enclosure.
  *Objective* Determine if mate choice in the pre-breeding season predicts reproductive success outcomes.
2. *Howdy socialization* Females were allowed access to one male through the howdy door prior to the breeding season (December–April) while the other neighboring male had a blocked howdy door. Behavioral observations were recorded from 1300 to 1400 h daily in 2018 and 1300–1400 h on Mondays, Wednesdays, and Fridays in 2019. Behaviors were averaged across all days for analysis.
  *Objective* Determine if socialization alters choice and affects reproductive success outcomes.
3. *Post-socialization dichotomous choice trial* One female between two males scored for a single day (0700 to 0700 the following morning) with all howdy doors open on both sides of the enclosure.
  *Objective* Determine if socialization alters mate preference.
4. *Breeding season pairing (May–June)* One female and one male allowed continuous access for breeding (see detailed description below). Behavioral observations were recorded from 1300 to 1400 h daily in 2018 and 1300–1400 on Mondays, Wednesdays, and Fridays in 2019. Behaviors were averaged across all days for analysis.
  *Objective* Determine if socialization and/or mate preference affect reproductive behaviors.

We designated preference in both the pre-socialization and post-socialization dichotomous choice tests by obtaining the percentage of behaviors females performed toward each neighboring male out of total behaviors performed during the trial for the following behavioral categories: conspecific investigation, breeding behavior, nesting behavior, olfactory communication behavior, social display behavior, and basking behavior. Males and females were designated as preferred if the focal animal performed more than 50% of their behaviors toward that male/female and the other male/female was designated as non-preferred. Pairs were additionally scored as mutually preferred if both the male and female had chosen each other as preferred (Preferred = Male_Preferred_ − Female_Preferred_, N = 7) and as mixed preference if either the male or female did not prefer the opposite sex conspecific (Mixed = either Male_Preferred_ − Female_Nonpreferred_ or Male_Nonpreferred_ − Female_Preferred_, N = 6) and mutually nonpreferred if neither preferred the opposite sex (Nonpreferred = Male_Nonpreferred_ − Female_Nonpreferred_, N = 11) resulting in three experimental groups.

### Breeding introduction and reproductive success measurements

Mate dyads were allowed continuous access for breeding during the breeding season (May–July) for mating introductions. Mate dyads used for the dichotomous choice tests were governed by the top two genetic recommendations (except in two instances, see below) from the Association of Zoos and Aquariums Species Survival Plan (SSP). Only animals recommended by the SSP were introduced for breeding, thus all pairings adhered to the SSP.

Howdy doors between the adjacent enclosures were opened to introduce the male and female for mating between 0900 and 1100 h. If either animal’s behavior reached contact aggression level two (see Supplemental Table 1), animal care staff immediately separated animals to their respective enclosures to prevent injury or death regardless of whether all three days had been reached. Mating pairs were introduced for three days with breeding access and video collection, then separated for 3–5 days. Males were alternated every other breeding cycle (after the 3–5 days of separation) with the first male for introduction randomized across the breeding pairs based on howdy door access. We also recorded and analyzed the behaviors for the total number of days each mate pairing was placed together for breeding. We ceased breeding introductions for the season when females started to show signs of nesting. Upon pair separation the Howdy Socialization door configurations were reinstated. When the animals were allowed outside, they had visual and chemical access to their neighbors, so they were kept inside until the breeding season pairings had ended (end of August). There were no recordings from 6/8/2019–6/18/2019 to 6/21/2019–7/8/2019 due to recorder malfunctions.

We monitored the following variables indicative of reproductive performance for all occurrences across the entire period animals were introduced for breeding:

1. whether successful copulation was seen (“Intromission Success” 1,0), and;
2. how many copulations were achieved throughout the breeding season (“number of copulations”);
3. the average number of copulations recorded across all mating introductions (“avg. number of copulation”);
4. the average copulation time per mounting in minutes (“avg. copulation time”);
5. whether egg production occurred (“egg production” 1,0);
6. the average number of eggs produced across the two different experimental groups (i.e. socialized and not socialized, preferred and non-preferred; “avg. number of eggs”).

### Data analysis

#### Effects of socialization on reproductive success and breeding behavior

All analyses were performed using R studio^55^ using R version 4.0.1 GUI 1.72 Catalina. To evaluate whether socialization affected reproductive performance, we used the *lme4* package in Bates et al.^56^. We ran GLMMs to examine the relationship between our socialization explanatory variable (1,0) and the reproductive performance variables Intromission success, average number of copulations, average copulation time, egg production, and average number of eggs. Socialization information for the focal pairing was matched to the reproductive success and breeding season mate introductions behavior data collected for that same year. We included male ID and female ID as random factors to account for repeated animals across years and variation in reproductive success across animals. Gaussian regression was used for the number of copulations, average number of copulations, and average copulation time. A poisson distribution was used for average number of eggs. Logistic regression with a logit-link function was used for intromission success and egg production. Snout-vent length (SVL) is correlated with clutch size in *Cyclura*^2^, thus, we tested the difference in female SVL between the socialized and non-socialized group using a GLMM with female SVL as the response variable, socialization group as the explanatory variable, and female ID as a random factor.

To investigate how breeding behaviors were affected by socialization, we subsetted behavioral data to the breeding season (May–June) during mate introductions. We grouped our full behavioral ethogram into functional behavioral categories based on knowledge of rock iguana behaviors from experienced keeper staff and literature review^2,20^. We summed the frequencies of the individual behaviors (Supplemental Table 1) resulting in the following behavioral categories for analysis: social display, olfactory communication, feed, basking, and resting. We also evaluated the individual behavior “Proximity” because the summed behavioral category of “conspecific investigation” used for mate preference designation was not meaningful for breeding behaviors since it included howdy-door specific behaviors which are not seen when animals are paired (Supplemental Table 1). We ran GLMMs assuming a negative distribution using the *glm nb* function from the MASS package^57^ to examine the relationship between socialization opportunities and our summed behaviors from the breeding season. We included focal ID as a random factor to account for variation in reproductive success across animals.

#### Effects of mate preference on reproductive success and breeding behavior

To evaluate whether mate preference affected reproductive performance, we used the *lme4* package in R^56^. We ran GLMMs to examine the relationship between both the male and female pre-socialization preference explanatory variable (Preference 1,0) and the mutual mate preference explanatory variable (preferred, mixed, nonpreferred) versus the reproductive performance variables intromission success, average number of copulations, average copulation time, egg production, and average number of eggs. Preference information for the focal pairing was matched to the reproductive success and breeding season mate introductions behavior data collected for that same year. We included male ID and female ID as random factors to account for repeated animals across years and variation in reproductive success across animals. Gaussian regression was used for the number of copulations, average number of copulations, and average copulation time. A poisson distribution was used for average number of eggs. Logistic regression with a logit-link function was used for intromission success and egg production. Snout-vent length (SVL) is correlated with clutch size in *Cyclura*^2^, thus, we tested the difference in female SVL between the preferred and non-preferred groups using a GLMM with female SVL as the response variable, socialization group as the explanatory variable, and female ID as a random factor.

Similar to our analyses on socialization, to investigate how preference impacted breeding behaviors, we used the summed frequency behavioral categories: social display, olfactory communication, feed, basking, and resting. We also evaluated the individual behavior proximity. Behavioral data was subsetted to the breeding season during mate introductions. We ran GLMMs assuming a negative distribution using the *glm.nb* function from the MASS package^57^ to examine the relationship between mate preference designations and our summed behaviors from the breeding season. We included focal ID as a random factor to account for variation in reproductive success across animals.

#### Mate preference and consistency between pre- and post-socialization mate choice

We assessed whether preference changed after the howdy socializations, to see if having contact with a potential mate was likely to influence a female’s preference in the post-socialization test. To do this, we compared whether focal females or males preferred the same males or females, respectively, before versus after socializations with a binomial test, set to a 50% probability of retaining the same preference.

#### The importance of socialization versus preference

We assessed whether the socialization or mutual mate preference variable was more important for predicting reproductive success by including both as explanatory variables in a GLMM with male and female ID included as random factors. We evaluated the following reproductive performance variables: intromission success, average number of copulations, average copulation time, egg production, and average number of eggs. Study design, analysis and reporting were carried out in accordance with the ARRIVE guidelines^58^.

### Ethical note

All methods were approved by the Institutional Animal Care and Use Committee (IACUC) of the San Diego Zoo Wildlife Alliance and deemed exempt as the cameras were hidden from the view of the animals and did not alter the environment or behavior of the animals. The animal facility was inspected by IACUC biannually. All husbandry practices were consistent with guidelines of the Association of Zoos and Aquariums (AZA) and the Husbandry Manual for West Indian iguanas^59^.

### Supplementary Information

Supplementary Table 1.

## Data Availability

Data for this project is available at the following link https://data.mendeley.com/datasets/j27x8v9cf7/1.

## References

[1] Case TJ, Bolger DT (1991). The role of introduced species in shaping the distribution and abundance of island reptiles. Evol. Ecol..

[2] Lemm JM, Alberts AC (2012). Cyclura: Natural History, Husbandry, and Conservation of West Indian Rock Iguanas.

[3] Wilson B (2016). The Jamaican iguana (*Cyclura collei*): A report of 25 years of conservation effort. Herp. Conserv. Biol..

[4] Alberts AC (1993). Chemical and behavioral studies of femoral gland secretions in iguanid lizards. Brain Behav. Evol..

[5] Bissell AN, Martins EP, Alberts AC, Carter RL, Hayes WK, Martins EP (2004). Behavior and ecology of Rock Iguanas, II. Iguanas: Biology and Conservation.

[6] Martins EP, Lacy KE, Alberts AC, Carter RL, Hayes WK, Martins EP (2014). Behavior and ecology of rock iguanas. Iguanas: Biology and Conservation.

[7] Cooper WE (1985). Female residency and courtship intensity in a territorial lizard, *Holbrooki propinqua*. Amph. Rep..

[8] Tokarz RR (1992). Male mating preference for unfamiliar females in the lizard, *Anolis sagrei*. Anim. Behav..

[9] Orrell KS, Jenssen TA (2002). Male mate choice by the lizard, *Anolis carolinensis*: A preference for novel females. Anim. Behav..

[10] Shine R, Webb JK, Lane A, Mason RT (2012). Familiarity with a female does not affect a male’s courtship intensity in garter snakes *Thamnophis sirtalis* parietalis. Curr. Zool..

[11] Leu ST, Burzacott D, Whiting MJ, Bull CM (2015). Mate familiarity affects pairing behavior in a long-term monogamous lizard: Evidence from detailed nio-logging and a 31-year field study. Ethology.

[12] Martin MS, Shepherdson DJ (2012). Role of familiarity and preference in reproductive success in ex situ breeding programs. Conserv. Biol..

[13] Martin-Wintle MS, Shepherdson D, Zhang G, Zhang H, Li D, Zhou X, Swaisgood RR (2015). Free mate choice enhances conservation breeding in the endangered giant panda. Nat. Commun..

[14] Drickamer L, Gowaty P, Holmes C (2000). Free female mate choice in house mice affects reproductive success and offspring viability and performance. Anim. Behav..

[15] Drickamer LC, Gowaty PA, Wagner DM (2003). Free mutual mate preferences in house mice affect reproductive success and offspring performance. Anim. Behav..

[16] Gowaty PA, Drickamer LC, Schmid-Holmes S (2003). Male house mice produce fewer offspring with lower viability and poorer performance when mated with females they do not prefer. Anim. Behav..

[17] Bluhm CK, Gowaty PA (2004). Social constraints on female mate preferences in mallards, *Anas platyrhynchos*, decrease offspring viability and mother productivity. Anim. Behav..

[18] Ihle M, Kempenaers B, Forstmeier W (2015). Fitness benefits of mate choice for compatibility in a socially monogamous species. PLoS Biol..

[19] Wiewandt, T. A. *Ecology, behavior, and management of the Mona Island Ground Iguana, Cyclura stejnegeri*. (Doctoral dissertation). Cornell University (1977).

[20] Iverson JB (1979). Behavior and ecology of the rock iguana *Cyclura carinata*. Bull. Fla. State Mus. Biol. Sci..

[21] Darwin C (1871). The Descent of Man, and Selection in Relation to Sex.

[22] Werner DI, Burghardt GM, Rand AS (1982). Social Organization and ecology of Land Iguanas, *Conolophus subcristatus*, on Isla Fernandina, Galapagos. Iguanas of the World: Their Behavior, Ecology, and Conservation.

[23] Pratt NC, Alberts AC, Fulton-Medler KG, Phillips JA (1992). Behavioral, physiological, and morphological components of dominance and mate attraction in male green iguanas. Zoo Biol..

[24] Gier, P. J. *Iguanid Mating Systems: Ecological Causes of Sexual Selection Consequences. (Doctoral dissertation)*. University of Oklahoma (1997).

[25] Wikelski M, Carbone C, Bednekoff PA, Choudhury S, Tebbich S (2001). Why is female choice not unanimous? Insights from costly mate sampling in marine iguanas. Ethology.

[26] Alberts AC, Lemm JM, Perry AM, Morici LA, Phillips JA (2002). Temporary alteration of local social structure in a threatened population of Cuban iguanas (*Cyclura nubila*). Behav. Ecol. Sociobiol..

[27] Moss JB, Gerber GP, Schwirian A, Jackson AC, Welch M (2019). Evidence for dominant males but not choosy females in an insular rock iguana. Behav. Ecol..

[28] Martins EP, Lamont J (1998). Estimating ancestral states of a communicative display: A comparative study of *Cyclura* rock iguanas. Anim. Behav..

[29] Dugan B, Wiewandt TV, Burghardt GM, Rand AS (1982). Socio-ecological determinants of mating strategies in iguanine lizards. Iguanas of the World: Their Behavior, Ecology, and Conservation.

[30] Hayes WK, Carter RL, Cyril S, Thornton B, Alberts AC, Carter RL, Hayes WK, Martins EP (2004). Conservation of an endangered Bahamian rock iguana I. Population assessments, habitat restoration, and behavioral ecology. Iguanas: Biology and Conservation.

[31] Pederson JM (1992). Field observations on the role of tongue extrusion in the social behavior of the desert iguana (*Dipsosaurus dorsalis*). J. Compar. Psychol..

[32] Lemm JM, Steward SW, Schmidt TF (2005). Reproduction of the critically endangered Anegada Island iguana. Int. Zoo Yearb..

[33] Browne RK, Wolfram K, García G, Bagaturov MF, Pereboom JJM (2011). Zoo-based amphibian research and conservation breeding programs. Amphib. Reptile Conserv..

[34] Mallinson JJ (1995). Conservation breeding programmes: An important ingredient for species survival. Biodivers. Conserv..

[35] Tribe A, Booth R (2003). Assessing the role of zoos in wildlife conservation. Hum. Dimens. Wildl..

[36] Farquharson KA, Hogg CJ, Belov K, Grueber CE (2020). Deciphering genetic mate choice: Not so simple in group-housed conservation breeding programs. Evol. Appl..

[37] Romero-Diaz C, Gonzalez-Jimena V, Fitze PS (2019). Corticosterone mediated mate choice affects female mating reluctance and reproductive success. Horm. Behav..

[38] Ehrig, R. W. The captive husbandry and propagation of the Cuban rock iguana, *Cyclura nubila*. In *AAZPA Reg. Conf. Proc.* 268–273 (1993).

[39] Senar JC, Mateos-Gonzalez F, Uribe F, Arroyo L (2013). Familiarity adds to attractiveness in matters of siskin mate choice. Proc. R. Soc. B.

[40] Aragón P, López P, Martín J (2001). Chemosensory discrimination of familiar and unfamiliar conspecifics by lizards: Implications of field spatial relationships between males. Behav. Ecol. Sociobiol..

[41] Rodda G (1992). The mating behavior of *Iguana iguana*. Smithson. Contrib. Zool..

[42] Swierk L, Myers A, Langkilde T (2013). Male mate preference is influenced by both female behaviour and morphology. Anim. Behav..

[43] Vitousek MN (2009). Investment in mate choice depends on resource availability in female Galápagos marine iguanas (*Amblyrhynchus cristatus*). Behav. Ecol. Sociobiol..

[44] Teyssier A, Bestion E, Richard M, Cote J (2014). Partners’ personality types and mate preference: Predation risk matters. Behav. Ecol. Sociobiol..

[45] Lopez P, Aragon P, Martin J (2003). Responses of female lizards, *Lacerta monticola*, to males’ chemical cues reflect their mating preference for older males. Behav. Ecol. Sociobiol..

[46] Olsson M, Madsen T (1995). Female choice on male quantitative traits in lizards—Why is it so rare?. Behav. Ecol. Sociobiol..

[47] Huyghen K, Vanhooydonck B, Herrel A, Tadic Z, Van Damme R (2011). Female lizards ignore the sweet scent of success: Male characteristics implicated in female mate preference. Zoology.

[48] Sullivan BK, Kwiatkowski MA (2007). Courtship displays in anurans and lizards: Theoretical and empirical contributions to our understanding of costs and selection on males due to female choice. Funct. Ecol..

[49] Martin J, Lopez P, Martín J, López P, Rheubert JL, Siegel DS, Trauth SE (2014). Pheromones and chemical communication in lizards. Reproductive Biology and Phylogeny of Lizards and Tuatara.

[50] Alonzo SH, Sinervo B (2001). Mate choice games, context-dependent good genes, and genetic cycles in the side-blotched lizard, *Uta stansburiana*. Behav. Ecol. Sociobiol..

[51] Martin-Wintle MS, Wintle JP, Diez-Leon M, Swaisgood RR, Asa CS (2019). Improving the sustainability of ex situ populations with mate choice. Zoo Biol..

[52] Vitousek MN, Rubenstein DR, Nelson KN, Wikelski M (2008). Are hotshots always hot? A longitudinal study of hormones, behavior, and reproductive success in male marine iguanas. Gen. Comp. Endocrinol..

[53] Candolin U (2003). The use of multiple cues in mate choice. Biol. Rev. Camb. Philos. Soc..

[54] Hudson RD, Alberts AC, Alberts AC, Carter RL, Hayes WK, Martins EP (2004). The role of zoos in the conservation of West Indian Iguanas. Iguanas: Biology and Conservation.

[55] *RStudio 2022.02.3+492 “Prairie Trillium” Release (1db809b8323ba0a87c148d16eb84efe39a8e7785, 2022-05-16) for macOS Mozilla/5.0 (Macintosh; Intel Mac OS X 12_4_0) AppleWebKit/537.36 (KHTML, like Gecko) QtWebEngine/5.12.10 Chrome/69.0.3497.128 Safari/537.36*.

[56] Bates D, Mächler M, Bolker B, Walker S (2015). Fitting linear mixed-effects models using lme4. J. Sat. Softw..

[57] Venables WN, Ripley BD (2002). Modern Applied Statistics with S.

[58] du Sert NP (2020). The ARRIVE guidelines 2.0: Updated guidelines for reporting animal research. J. Cereb. Blood Flow Metall..

[59] Lemm, J.M., Lung, N. and Ward, A.M., 2010. Husbandry manual for West indian iguanas. http://www.iucn-isg.org/wp-content/uploads/2013/03/West_Indian_Iguana_Husbandry_Manual.pdf. Accessed 17 Nov 2023.

