# Supplementary material for: Social opportunities and mate preference improve breeding success in Caribbean iguanas
Source: Sci Rep. 2023 Nov 27;13:20877. doi: 10.1038/s41598-023-47599-3 (PMC10682467; doi:10.1038/s41598-023-47599-3)
Supplement: Supplementary file 1 — Supplementary Table 1. [file 41598_2023_47599_MOESM1_ESM.docx]

**Supplemental Material**

*Title:* Social opportunities and mate preference improve breeding success in Caribbean iguanas

***Jeffrey M. Lemm^1^, Meghan S. Martin^2^

^1^Conservation Science and Wildlife Health, San Diego Zoo Wildlife Alliance, 15600 San Pasqual Valley Rd., Escondido, California 92027 USA *jlemm@sdzwa.org*

^2^PDXWildlife, 5223 SE 41st Ave., Portland, OR 97202 USA *pdx@pdxwildlife.com*

Supplemental Table 1. Caribbean iguana behavioral ethogram. Gray rows indicate behaviors used in analysis that were either summed from the larger ethogram into functional categories or stand-alone behaviors.

| **Behavior** | **Definition** |
| --- | --- |
| Conspecific Investigation | The sum of the following behaviors |
| Investigating "Howdy Door" (HD) | Animals is at howdy door or in very close proximity to it (less than 12 inches/30cm) |
| Scratching Howdy Door | Animal is actively scratching at the howdy door |
| Walk | Short bout of directional travel between points, or sustained locomotion in a non-stereotyped manner. |
| Investigative Walking | Animal is slowly walking around enclosure, often tongue-flicking as if searching for something; no apparent path (as opposed to a straight definitive walk e.g. from BUV to food) |
| <1 Body Length Proximity | Animal is within at least one body length (~1m) of another animal. Behavior is non-mutually exclusive so should be turned "on" when animals are within one body length and all other behaviors should receive the modifier of -P while they remain within <1 body length. Includes when animals are separated by open or closed howdy door. |
| Breeding Behavior | The sum of the following behaviors |
| Copulation Attempt | Male climbs on female's back and the cloacal regions are brought into contact or near contact. May grab the female's nuchal crest in the mouth and grasp the sides. |
| Intromission | Tails are wrapped, hemipenis is successfully inserted into cloaca. Most activity ceases and animals remain relatively still. |
| Female Breeding Presentation | Female lowers body and lifts vent and tail. Often will try to get underneath the male. |
| Male Courtship Display | Male headbobs, often in a wobbly side-to-side motion or fast with a low amplitude, sometimes with mouth open slightly; usually while walking toward a female or around her enclosure. |
| Submissive Posturing | An animal (usually female) lies with body close to ground and head down when in close proximity to a conspecific (usually male). Tail is often slightly raised. |
| Nesting Behaviors | The sum of the following behaviors |
| Burrowing/ Digging | Digging with front and hind limbs, often short in duration (under 5 minutes). This is typically in corners or at existing burrows |
| Nest Guarding | Female within close proximity (~2m) to the nest site. Stays close for up to two weeks and usually shows aggression towards keepers if they try to approach the nest. |
| Nesting (only females) | Digging large burrow in substrate. Often performed at night. This behavior is distinguished from normal burrowing/digging by the longer duration in the same burrow and/or staying in the burrow for an extended period. Females will usually cover the burrow after digging. |
| Olfactory Communication | The sum of the following behaviors |
| Femoral Pore Drag | Scent marking with underside of rear thighs by dragging the thighs across a surface. |
| Face Rub | When an animal rubs their face/head on an object, ground, or other animal. Possibly a type of scent marking |
| Vent Drag | Scent marking with cloaca by dragging cloaca across a surface. |
| Tongue Flicking | Tongue is touched to any surface or animal |
| Defecate | Passing of fecal matter. |
| Contact Aggression | The sum of the following behaviors |
| Contact Aggression, Level 1 | A non-sustained (i.e., less than 5 seconds) aggressive interaction of moderate intensity, including actions such as head-bobbing (low amplitude/fast), gaping, high-walking, laterally compressed body, turning sideways to the other animal, charging, lunging, face-to-face interactions with short charges, nipping at tail and limbs**.** “Moderate intensity” is defined as aggressive acts which have low potential for inflicting serious injury, e.g., drawing blood. Generally accompanied by behaviors above and chasing, with bites to limbs and other parts of the body and/or short tail whips |
| Contact Aggression, Level 2 | Sustained aggression, including vigorous and potentially injurious physical contact (fighting, attacking), such as those in N2 and C1, yet more intense head-bobbing, face-to-face interactions, jaw-wrestling, biting for longer periods, biting onto a limb and rolling, chasing with contact. Intensity of aggression is high, will often have injury |
| Tail Whipping | tail is used to hit another animal with high intensity/speed |
| Biting | One animal either nips or gives a full bite to another animal |
| Jaw wrestling | When two animals are in a bout of aggression and both animals have jaws wide open, pushing one another (usually jaw to jaw). Animals can be facing each other (nose to nose), side by side with bodies parallel to one another or with bodies facing each other with heads at one another's shoulder area |
| Social Display Behavior | The sum of the following behaviors |
| Face to Face Confrontation | Animals are facing each other, usually with heads on the ground (often while gaping hissing, or doing short lunges at one another) |
| Non- Contact Aggression, Level 1 | Agitated. Interaction which includes head-bobbing (often low amplitude/fast) and gaping |
| Non- Contact Aggression, Level 2 | Threatening. Interaction which includes high-walking, laterally compressed body, hissing, head-bobbing (low amplitude/fast), gaping, lunging |
| Headbob | Moves head up and down in a vertical plane. Modifiers: Low Amplitude = movement of head does not go beyond 1/2 head depth, High Amplitude=movement of head is greater than or equal to 1/2 head depth, Quick, Slow; Gape (G)=Animal has mouth open, often during headbobbing - mouth can barely be open or be open very wide. Often during aggressive encounters |
| Gape | Animal has mouth open, often during headbobbing - mouth can barely be open or be open very wide. Often during aggressive encounters |
| Tail wagging | Usually during an aggressive encounter (sometimes during breeding attempts) - tail is low to the ground and proximal tail tip wags back and forth in a side to side movement |
| High-Walking | Male and female extend legs as far as possible while walking. Body laterally compressed. |
| Yawning | Animal is usually sitting still and mouth is opened slowly all the way to full extension |
| Hissing | A loud expression of air from the lungs |
| Feed | The sum of the following behaviors |
| Drinking | Drinking water |
| Eating | Feeding on provisioned food or planted plants |
| Resting Behaviors | The sum of the following behaviors |
| Sleep | Lying or sitting down asleep with eyes closed inside or outside a burrow. |
| In Burrow | Animal in burrow |
| Resting | Lying or sitting down with no to little movement, but awake with eyes open. |
| Basking | Basking under either ceramic heaters (no light given off), UV/heat bulbs (with visible light spectrum), or natural light |
| Keeper Interactions | Keeper staff are in enclosure. Animals may or may not interact with keeper, but animals may have behavioral changes when keepers are in enclosure and shortly thereafter |
| Not Visible | Animal is temporarily out of view within the indoor enclosure or is in outside enclosure with no camera view. |
| Other | Other behavior not described above |
